# Insights into non-Fickian solute transport in carbonates

**DOI:** 10.1002/wrcr.20238

**Published:** 2013-05-28

**Authors:** Branko Bijeljic, Peyman Mostaghimi, Martin J Blunt

**Affiliations:** 1 Department of Earth Science and Engineering, Imperial College LondonLondon, UK

## Abstract

[1] We study and explain the origin of early breakthrough and long tailing plume behavior by simulating solute transport through 3-D X-ray images of six different carbonate rock samples, representing geological media with a high degree of pore-scale complexity. A Stokes solver is employed to compute the flow field, and the particles are then transported along streamlines to represent advection, while the random walk method is used to model diffusion. We compute the propagators (concentration versus displacement) for a range of Peclet numbers (*Pe*) and relate it to the velocity distribution obtained directly on the images. There is a very wide distribution of velocity that quantifies the impact of pore structure on transport. In samples with a relatively narrow spread of velocities, transport is characterized by a small immobile concentration peak, representing essentially stagnant portions of the pore space, and a dominant secondary peak of mobile solute moving at approximately the average flow speed. On the other hand, in carbonates with a wider velocity distribution, there is a significant immobile peak concentration and an elongated tail of moving fluid. An increase in *Pe*, decreasing the relative impact of diffusion, leads to the faster formation of secondary mobile peak(s). This behavior indicates highly anomalous transport. The implications for modeling field-scale transport are discussed.

**Citation:** Bijeljic, B., P. Mostaghimi, and M. J. Blunt (2013), Insights into non-Fickian solute transport in carbonates, *Water Resour. Res.*, 49, 2714–2728, doi:10.1002/wrcr.20238.

## 1. Introduction

[2] Flow and solute transport play an important role in a number of applications in geological porous media, including storage of carbon dioxide, contaminant transport and the associated access to clean drinking water, safe disposal of nuclear waste, and enhanced oil recovery. Although carbonate formations contain more than half of the world’s conventional oil reserves [*Chilingar et al*., 1972; *Ahlbrandt et al*., 2005], the interplay of physical processes involving transport through their complex structures with heterogeneities from the pore scale upward is not fully understood.

[3] Experimental studies of transport behavior in carbonate rock in both the laboratory [*Baker*, 1977; *Bretz and Orr*, 1987; *Gist et al*., 1990; *Hidajat et al*., 2004; *Oshita and Okabe*, 2005; *Fourar et al*., 2005; *Fourar and Radilla*, 2009] and the field [*Cacas et al*., 1990; *Gelhar et al*., 1992, and references therein; *Maloszewski and Zuber*, 1993; *Meigs and Beauheim*, 2001; *Witthüser et al*., 2003; *Birk et al*., 2005; *Gouze et al*., 2008a] have typically found an early breakthrough of the solute and a long tailing of the concentration at late times. At the core scale, effluent breakthrough curves (BTCs) of a sucrose tracer injected into brine-saturated San Andres carbonate cores have shown a considerable degree of tailing due to significant core heterogeneity [*Bretz and Orr*, 1987]. *Gist et al*. [1990] associated longtime tails of NaCl brine tracer BTCs in heterogeneous carbonate rocks (including dolostone and a Middle Eastern carbonate) with macroscopic permeability heterogeneities on the millimeter-to-centimeter scale, in contrast to BTCs in less heterogeneous carbonates (including Austin chalk, Oolitic limestone, and Indiana limestone) that did not show pronounced tailing. *Hidajat et al*. [2004] measured both in situ (by X-ray computed tomography (CT) scanning) and outlet NaI tracer concentrations in vuggy carbonate samples from a west Texas field and observed a very early breakthrough followed by a long tail: this implied the existence of a sample-spanning high-permeability streak in a tight matrix. At the field scale, good examples of prolonged tailing of BTCs in carbonate rock can be found in the experimental studies of *Meigs and Beauheim* [2001], *Witthüser et al*. [2003], *Birk et al*. [2005], and *Gouze et al*. [2008a].

[4] This late-time behavior cannot be modeled by a deterministic advection-dispersion equation (employing Fick’s law at the macroscale) in a homogenous domain; more sophisticated theories are required, such as multirate mass transfer models [*Haggerty and Gorelick*, 1995; *Haggerty et al*., 2000] and continuous time random walks (CTRWs) [*Berkowitz et al*., 2006]. The review by *Berkowitz et al*. [2006] provides an excellent overview of these and other transport modeling approaches. Behavior that cannot be described by the advection-dispersion equation has been coined “anomalous,” or non-Fickian, and is very often encountered in complex geological media, from laboratory studies to the field scale [*Levy and Berkowitz*, 2003; *Becker and Shapiro*, 2003; *Gouze et al*., 2008a].

[5] Studying BTCs is very useful in assessing solute first arrival times. However, having an accurate description of plume concentration as a function of distance in either a core or at the field scale provides full information on the in situ transport processes. If the injected tracer particles rapidly fully sample the velocity field, the transport is Fickian and can be described by the advection-dispersion equation, resulting in a concentration profile whose peak moves at the average flow speed with a Gaussian spread: this is typical of homogeneous media where each particle encounters the relatively narrow range of flow speed after traveling through only a few pores, as shown for unconsolidated bead packs [e.g., *Scheven et al*., 2005]. However, in complex porous media, such as carbonate rock, the solute experiences a very wide range of transit times across pores of very different size; consequently the particle transport deviates from Fickian behavior, resulting in large variations of plume shape from a Gaussian profile, as discussed by *Berkowitz and Scher* [2001] and *Scher et al*. [2002].

[6] The transport can be described by a probability density function (PDF) of either the displacement or transit time of solute particles. PDFs have been studied experimentally by nuclear magnetic resonance (NMR) measurements where the distribution of displacement of moving protons is obtained [*Callaghan*, 1991; *Gladden*, 1994]; these are also called the NMR flow propagators [*Kärger and Heink*, 1983]. The propagators have been measured on consolidated rock cores in Fontainebleau sandstone [*Packer and Tessier*, 1996; *Tessier et al*., 1997; *Tessier and Packer*, 1998], Bentheimer sandstone [*Waggoner and Fukushima*, 1996; *Johns et al*., 2003; *Scheven et al*., 2005; *Verganelakis et al*., 2005; *Singer et al*., 2006; *Mitchell et al*., 2008a], Portland carbonate [*Scheven et al*., 2005; *Verganelakis et al*., 2005; *Mitchell et al*., 2008b], and a dolomite [*Zhao et al*., 2010]. A critical discussion of these measurements is presented by *Gladden and Mitchell* [2011]. These experiments clearly distinguish the nature of non-Fickian transport in a homogeneous bead pack from that in sandstones and even further, from that in carbonate rock. *Scheven et al*. [2005] have demonstrated that the propagators measured in a bead pack show a non-Gaussian shape only for a short time and then become Gaussian about the mean displacement; for Bentheimer sandstone a pronounced peak is observed representing the stagnant fluid regions that gradually disappears with time; for Portland carbonate the stagnant peak is both larger and more persistent than that for sandstones, implying a much greater degree of particle retardation.

[7] In this modeling study we compute PDFs of solute displacement for a suite of carbonate rock images over a wide range of Peclet numbers (*Pe* = *u*_av_*L*/*D_m_*, where *u*_av_ is the average flow speed, *L* is the characteristic length, and *D_m_* is the molecular diffusion coefficient) to demonstrate the nature of non-Fickian transport in different classes of carbonate. To describe advection and diffusion at the pore scale, random-walk-based particle tracking techniques have been a common choice, either simulating transport directly on the voxelized images of the pore space, or on extracted pore networks. Network modeling has been widely used for studying solute transport [*Saffman*, 1959,^1960^; *Sahimi et al*., 1986; *Sorbie and Clifford*, 1991; *Damion et al*., 2000; *Bruderer and Bernabe*, 2001; *Bijeljic et al*., 2004; *Picard and Frey*, 2007; *Acharya et al*., 2007; *Rhodes et al*., 2008]. Advection is solved analytically in a unit network bond, and random walk movement is superimposed to simulate diffusion. Advances have been made in the description of the asymptotic dispersion coefficients over a wide range of Peclet numbers [*Bijeljic et al*., 2004; *Acharya et al*., 2007] including an explanation for the power-law dependence of longitudinal dispersion coefficient as a function of *Pe*, reconciling experiment, pore-scale modeling, and CTRW theory for Berea sandstone [*Bijeljic and Blunt*, 2006; *Dentz et al*., 2004]. Propagators have been studied using network models representing Berea sandstone [*Picard and Frey*, 2007] and for a dolomite [*Zhao et al*., 2010]. The latter study has shown a good agreement with NMR experiments using an adjustable parameter to describe the pore dynamics.

[8] In parallel with network modeling, a number of approaches have been developed to simulate transport directly on a 3-D voxel representation of the porous medium obtained by direct X-ray (synchrotron or micro-CT) scanning or by reconstructing pore space from 2-D thin section images. The finite difference method has been used to compute flow in reconstructed Fontainebleau sandstone [*Salles et al*., 1993; *Tessier et al*., 1997; *Stapf et al*., 2000], reconstructed Vosges sandstone [*Yao et al*., 1997], reconstructed random spherical and aspherical packings [*Coelho et al*., 1997], micro-CT images of Berea and Bentheimer sandstones [*Bijeljic et al*., 2011a; *Mostaghimi et al*., 2012; *Blunt et al*., 2013; *Bijeljic et al*., 2013], and an image of Portland carbonate [*Bijeljic et al*., 2011a, 2013]. The finite element method was used on a model sand pack [*Cardenas*, 2008,^2009^], while the finite element/finite volume method was employed to compute flow in an image of Fontainebleau sandstone [*Zaretskiy et al*., 2010]. In addition, particle-based approaches have been used to find the flow field and simulate the transport of solute. The lattice-Boltzmann method has been employed to compute flow in computer model-generated bead packs [*Lowe and Frenkel*, 1996; *Maier et al*., 2000; *Kandhai et al*., 2002], directly on an NMR image of a spherical bead pack [*Manz et al*., 1999], and on an NMR image of a spherical bead pack that was modified to represent a Bentheimer sandstone core [*Johns et al*., 2003]. The modified moving particle semi-implicit method was used to compute dispersion through micro-CT images of Berea and two other sandstones [*Ovaysi and Piri*, 2011].

[9] Significant progress in describing Fickian and non-Fickian dispersion has been made in the studies that use direct transport simulation on the pore space. Findings on Fickian dispersion include description of the asymptotic dispersion coefficients over a wide range of Peclet numbers directly in the pore space of unconsolidated bead packs [*Coelho et al*., 1997; *Maier et al*., 2000] on sandstones [*Salles et al*., 1993; *Ovaysi and Piri*, 2011; *Bijeljic et al*., 2011a; *Mostaghimi et al*., 2012] and carbonate rock [*Bijeljic et al*., 2011a, 2013]. Non-Fickian dispersion results include agreement between direct pore-scale simulations and experimentally measured NMR propagators for bead packs [*Manz et al*., 1999; *Kandhai et al*., 2002; *Maier et al*., 2008], sandstones [*Tessier et al*., 1997; *Blunt et al*., 2013; *Bijeljic et al*., 2013], and a carbonate [*Bijeljic et al*., 2011a, 2013]. However, almost all of the abovementioned studies deal with bead packs, sand packs, and sandstones that have a narrower distribution of pore size giving, as shown later, a narrower spread of local velocities than in carbonates.

[10] Despite huge advances in computer power and algorithmic efficiency, studies of dispersion have, to date, been limited to relatively small samples. The Fontainebleau sandstone image used for mesh generation in the study by *Zaretskiy et al*. [2010] had 200^3^ voxels with a resolution of 7.5 µm giving an overall size of 1.5 × 1.5 × 1.5 mm^3^. *Ovaysi and Piri* [2011] used 42 × 42 × 190, 66 × 66 × 298, and 52 × 52 × 234 voxels for Berea and the two other studied sandstones, respectively. The corresponding image resolutions were 10.69 µm for Berea and 6.796 and 8.683 µm for the other two sandstones, resulting in sample sizes of approximately 0.45 mm in the *x* and *y* directions and 2.03 mm in the flow direction.

[11] In our previous work [*Bijeljic et al*., 2011a] we employed an efficient streamline-based algorithm with a random walk method to study solute dispersion on micro-CT images of a sand pack, Berea sandstone, and Portland limestone containing 300^3^ grid blocks (voxels) at a resolution (voxel size) of 10 µm, 5.345 µm, and 9 µm, respectively, representing a cube of side length 1.6–3.0 mm. The qualitatively different signature of transport through the major porous rock types encountered in the subsurface (sand packs, sandstones, and carbonates) was demonstrated. A very good agreement was found between NMR measurements [*Scheven et al*., 2005; *Mitchell et al*., 2008b] and the model results [*Bijeljic et al*., 2011a, 2013].

[12] However, while the connection between non-Fickian transport behavior as a result of a wide range of transit times has been made [*Berkowitz and Scher*, 2001; *Scher et al*., 2002; *Bijeljic and Blunt*, 2006], in this paper we provide a systematic study to describe the non-Fickian behavior arising from the relationship between the complex pore structure and velocity field to characterize transport in heterogeneous carbonates. To date, there have been no modeling studies performed directly on the images of carbonate rocks for a suite of samples and over a range of *Pe*: the aim of this work is to predict quantitatively the non-Fickian transport characteristics in carbonate rock of different structures and over a range of flow conditions. We study the nature of early breakthrough and long tailing plume behavior by simulating transport of a solute through 3-D X-ray images of six different carbonate rock samples, representing geological media with a high degree of pore-scale complexity. A Stokes solver is employed to compute the flow field, and the particles are then transported semianalytically along streamlines to represent advection, and the random walk motion is used to model diffusion. We describe the different non-Fickian transport behaviors in different types of carbonate by analyzing propagators (concentration versus displacement) for a wide range of Peclet numbers and explain this behavior by analyzing PDFs of the velocity distribution.

## 2. X-Ray Images and Mathematical Model

### 2.1. Images

**Figure 1:**
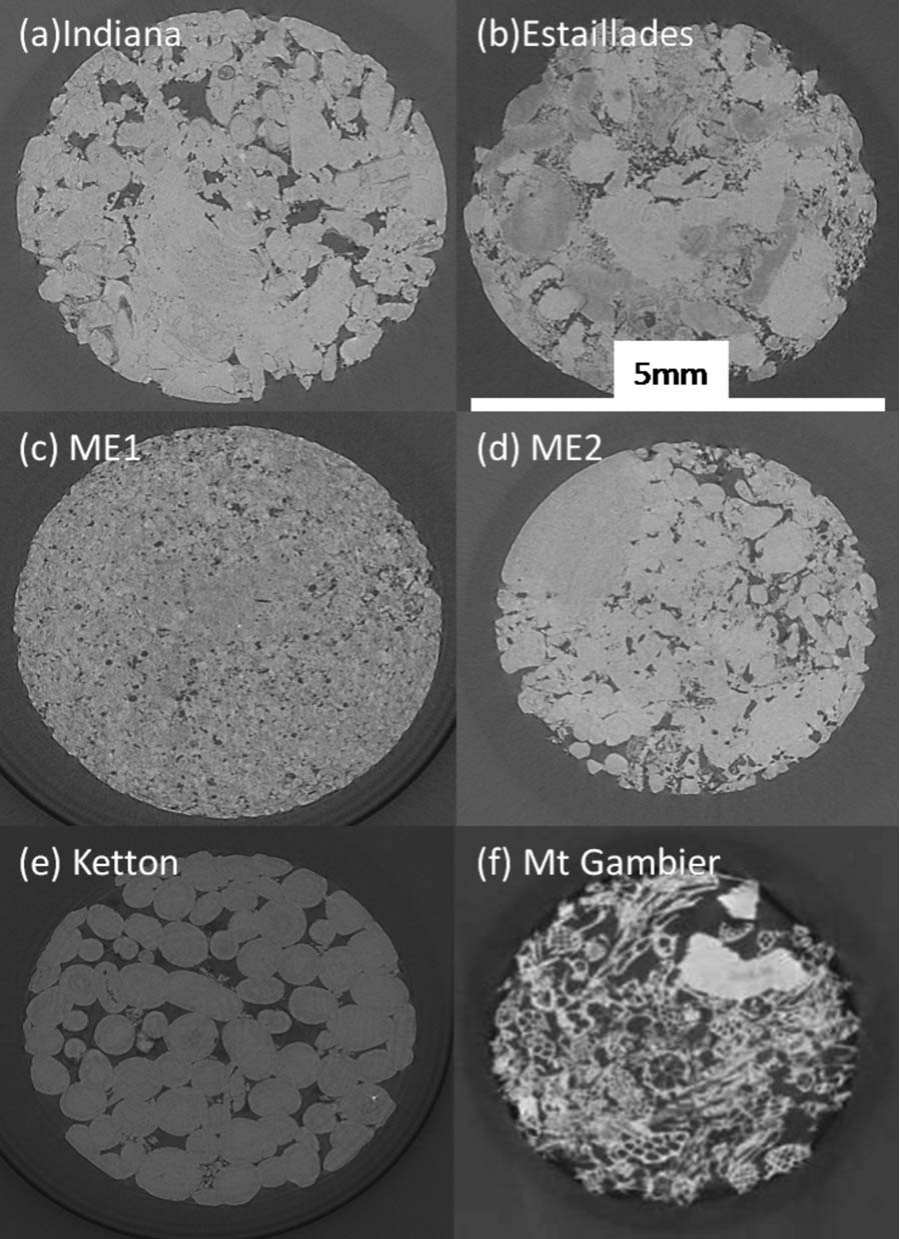
2-D cross sections of 3-D gray-scale images for the six carbonate rock samples studied: (a) Indiana limestone, (b) Estaillades limestone, (c) ME1, (d) ME2, (e) Ketton limestone, and (f) Mount Gambier limestone. The images were acquired with a SYRMEP beamline at the ELETTRA Synchrotron in Trieste, Italy.

[13] For transport studies we use four quarry carbonate samples (Indiana, Estaillades, Ketton, and Mount Gambier limestones) and two carbonate samples from a Middle East aquifer (denoted Middle Eastern carbonate 1 (ME1) and Middle Eastern carbonate 2 (ME2)). The dry scan images were acquired on cylindrical cores having 5 mm diameter and 25 mm length with a synchrotron beamline (Synchrotron Radiation MEdical Physics (SYRMEP) beamline at the ELETTRA Synchrotron in Trieste, Italy) at a resolution of 7.7 µm (for Indiana, Estaillades, Ketton, ME1, and ME2) and 9 µm (for Mount Gambier), corresponding to two different detector pixel sizes of 3.85 µm and 4.5 µm; the charge coupled device (CCD) camera binned the results giving the final voxel size of twice the detector pixel size. The range of energy used was 27–33 keV, and each scan lasted between 3 and 4 h. Reconstruction was performed by in-house software, resulting in images of around 600^3^ voxels from which a central cubic section was taken for our simulations. The 2-D cross sections of 3-D gray-scale images for the six carbonates studied are shown in Figures 1a–1f. Segmentation into binary images was based on a histogram analysis employing Otsu’s thresholding algorithm and using ImageJ software [*Sahoo et al*., 1988]. In addition, we acquired an additional image at a higher resolution, 3.3 µm voxel size, for Estaillades using a micro-CT scanner (Xradia Versa).

[14] The voxel size, number of voxels, system size, porosities, permeabilities, characteristic length, and average coordination number of the carbonate rocks studied are given in Table 1. The average coordination numbers are obtained by extracting pore networks from the images using the maximal ball algorithm [*Dong and Blunt*, 2009; *Gharbi and Blunt*, 2012]. The pore networks are a topological representation of the pore space as wide pores connected by throats. The coordination number is the number of throats connected to each pore. We define the characteristic length *L* (needed for calculating *Pe*) for each carbonate image based on a cubic packing of regular spheres. For this idealized system, the grain diameter is *π*V/S, where V is the volume of the porous medium (pore plus grain), and S is the area of the pore-solid interface. We use the same definition for our images since the volume and the pore-solid area are readily computed, while it is difficult to identify individual grains unambiguously. The image sizes are 320^3^−380^3^ voxels in total representing cubes of side length 2.46–3.15 mm, representing 8–43 characteristic lengths; the higher-resolution Estaillades image is 650^3^ voxels in total and has a side length of 2.1 mm.

**Table 1:** Description of the Seven Carbonate Images Studied, Including Voxel Size, Number of Voxels, Image Size, Porosity, Permeability, Characteristic Length Estimate, and Average Coordination Number

Sample	Voxel Size (µm)	Number of Voxels	1 D Size (µm)	Porosity	Permeability (mD)	Characteristic Length Estimate (µm))	Average Coordination Number
ME1	7.7	380^3^	2926	0.093	32	166.2	2.50
Indiana	7.7	330^3^	2541	0.110	292	299.6	2.97
Estaillades	7.7	350^3^	2695	0.133	328	158.2	3.03
Estaillades high resolution	3.3	650^3^	2145	0.118	492	243.2	3.32
Ketton	7.7	350^3^	2695	0.149	8476	320.7	3.08
ME2	7.7	320^3^	2464	0.175	1538	161.65	3.64
Mt. Gambier	9.0	350^3^	3150	0.556	16607	72.2	7.41

[15] Porosities are computed on the images from the ratio of number of pore voxels, *N*_pvox_, divided by total number of voxels, *N*_vox_. Voxels that are not connected to the inlet or outlet are excluded from the analysis and the flow calculations. It can be seen that the carbonates that have a low porosity tend to be poorly connected and have a lower permeability, provided that the characteristic length is similar.

### 2.2. Mercury Injection Capillary Pressure Curves

**Figure 2:**
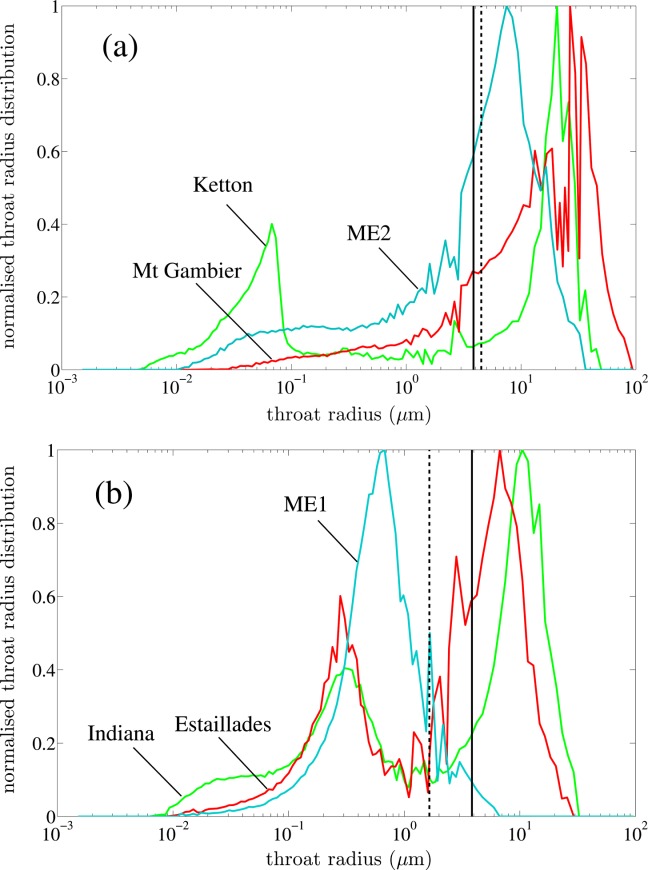
Throat radius distributions obtained from MICP measurements for (a) Ketton, Mt Gambier, and ME2 and (b) Indiana, Estaillades, and ME1. The straight black solid lines in (a) and (b) mark half the voxel size (3.85 µm) for images of Indiana, Estaillades, ME1, ME2, and Ketton. The dashed line in (b) marks half the voxel size (4.5 µm) for the image of Mt Gambier, while the dashed line in (a) marks half the voxel size (1.65 µm) for the high-resolution image of Estaillades. This is the smallest throat radius that can be detected in the images.

[16] Mercury injection capillary pressure (MICP) was measured at a commercial laboratory (Weatherford) on samples taken from the same block of stone from which the images were obtained. Figures 2a and 2b show the inferred throat radius distributions normalized to a maximum value obtained from MICP for (a) Ketton, Mt Gambier, and ME2 and (b) Estaillades, Indiana, and ME1. Plotted also are the straight black solid and dashed lines that mark half the voxel size of the images studied, representing the smallest throat radius that can be detected in the images.

[17] Carbonate rocks are, in general, characterized by a wide range of pore size and significant microporosity: pores less than 1 µm across. This microporosity is not imaged and hence not accounted for in our simulations. With the image resolution we had available, most of the macroporosity of the pore space is captured for Indiana, Estaillades, Ketton, Mt Gambier, and ME2, while less of the macropores are scanned for ME1. We will discuss later that consideration of microporosity would further emphasize the findings from this study associated with a large fraction of stagnant solute. A complete discussion of microporosity is beyond the scope of this paper and requires the acquisition of much higher-resolution images.

### 2.3. Flow Model

[18] Incompressible steady viscous flow is simulated directly through the pore-space images by solving the volume conservation equation (1) and the Navier-Stokes equation (2):

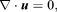
(1)

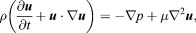
(2)
where ***u*** is the velocity vector, *μ* is viscosity of water (*μ* = 0.001 Pa s), *ρ* is the density of water (*ρ* = 1000 kg/m^3^), and *p* is the pressure. We use a standard finite volume method implemented in *OpenFOAM* [2011]. The pressure and velocity are solved iteratively based on the pressure implicit with splitting of operators (PISO) algorithm of *Issa* [1986] (see *Raeini et al*. [2012] for further details).

[19] The simulations are run at a *Re* = *ρu*_av_*L*/*µ* ≪ 1 assuming a steady state ∂*u*/∂*t* = 0. This means that slow flow is simulated; the second term on the left in equation (2) is small compared to the second term on the right (viscous) term. The average flow speed is calculated as *u*_av_ = *q*/*ε*, where *q* = *Q*/*L_y_L_z_* is the Darcy velocity, *Q* (m^3^/s) is the total volumetric flux calculated as 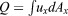, where 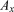 (m^2^) is the cross-sectional area of void voxels perpendicular to the direction of flow *x*, and *u_x_* is the face velocity that is normal to 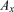; *L_x_*, *L_y_*, and *L_z_* are the image lengths in each direction, and *ε* is the porosity. Each voxel in the image is converted to a grid block in the finite volume mesh.

[20] The flow domain is cubic. We use constant pressure boundary conditions for pressure at the left and the right faces of the images (the pressure drop is Δ*P*). For the other faces of the images and for the solid walls, no-flow boundary conditions are used. We obtain the velocities and pressures for each voxel and calculate absolute permeability *k* (m^2^) from Darcy’s law:

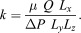
(3)

[21] The permeability values in Table1 are given in mD, where 1 mD = 9.869233 × 10^−16^ m^2^.

**Figure 3:**
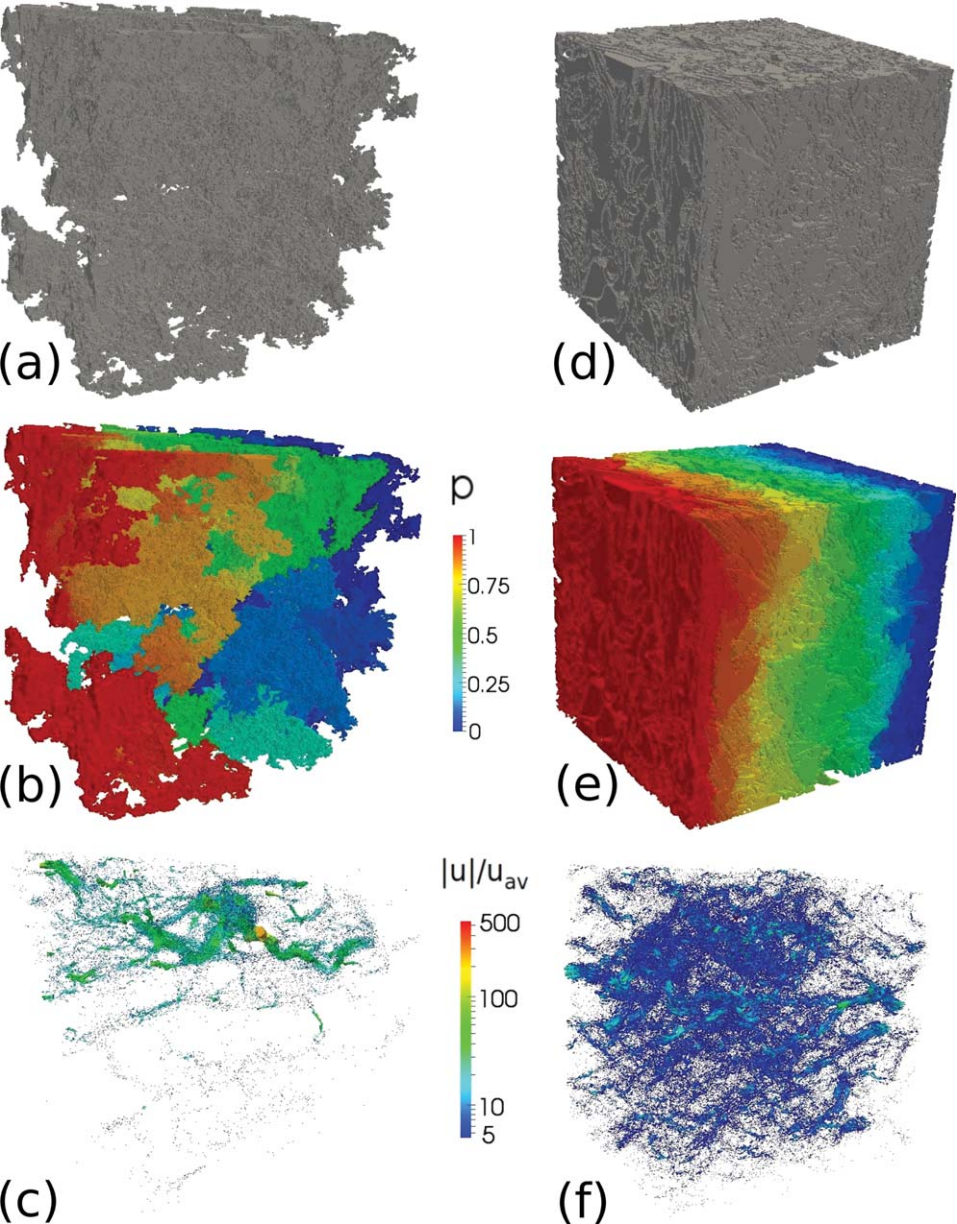
Estaillades limestone image: (a) the geometry shown as the pore volume represented by gray color; (b) normalized pressure field with a unit pressure difference across the model; (c) normalized flow field, where the ratios of the magnitude of *u* at the voxel centers divided by the average flow speed *u*_av_ are shown using cones that are colored using a logarithmic scale spanning from 5 to 500. The same figures are shown for the Mount Gambier limestone image, denoted as (d), (e), and (f), respectively.

[22] An illustration of how flow is computed on the synchrotron images of Estaillades limestone (that is an exemplar for a carbonate with a wide spread of velocities) and Mount Gambier limestone (that is an exemplar for a carbonate with a narrower spread of velocities) is presented in Figures 3a–3f. The pore geometry, pressure field, and velocity field are shown. The velocity field figures show a subset of pore voxels where advection is dominant in comparison to diffusion: the stagnant flow voxels are not represented in Figure 3.

**Figure 4:**
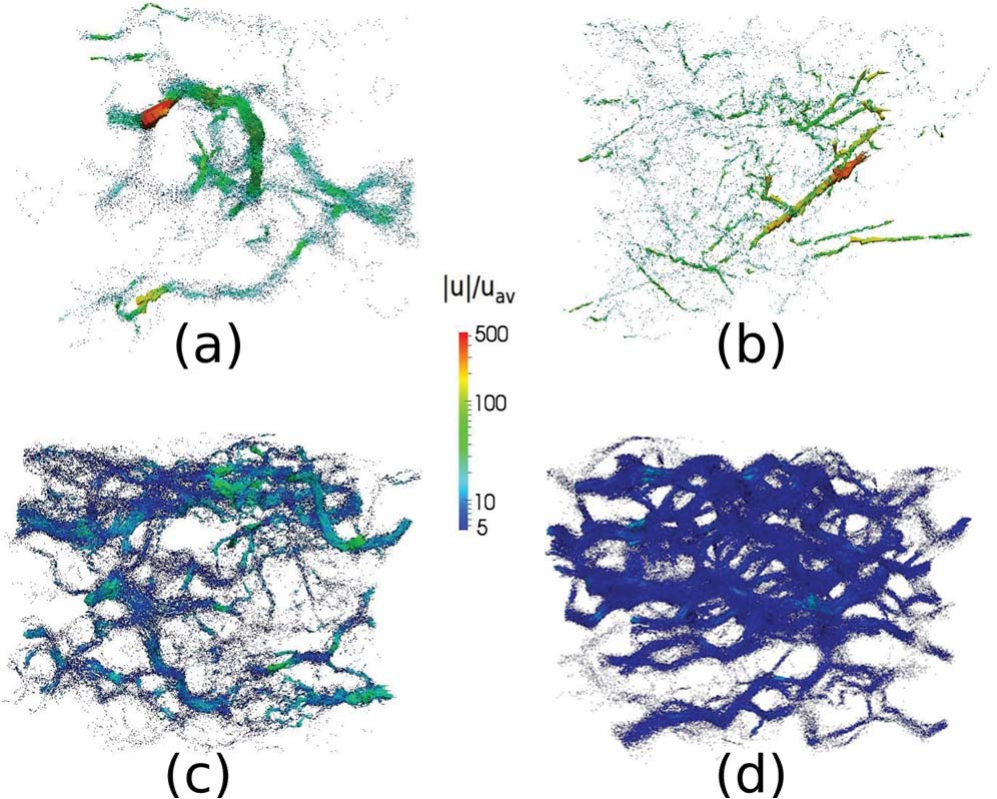
Normalized flow fields for (a) Indiana limestone, (b) ME1, (c) ME2, and (d) Ketton limestone. Again, the ratios of the magnitude of *u* at the voxel centers divided by the average flow speed *u*_av_ are shown using cones that are colored using logarithmic scale spanning from 5 to 500.

[23] Figures 3c and 3f show the very different nature of the velocity fields: while in the low-connectivity Estaillades limestone flow is concentrated in a few channels with much of the pore space largely stagnant, in the highly connected Mount Gambier limestone flow is evenly distributed throughout the sample and is characterized by less tortuous channels. Qualitatively similar flow fields to that of Estaillades limestone can be seen in Figures 4a and 4b for Indiana limestone and ME1. Figure 4d represents the flow field in Ketton limestone that is qualitatively similar to that of Mt Gambier and ME2 (Figure 4c). This will be discussed in more detail later.

**Figure 5:**
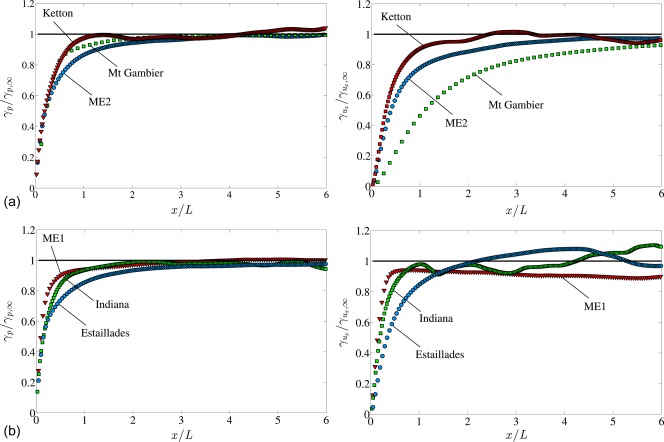
Variograms showing the normalized functions for porosity,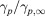, and velocity in the direction of flow, 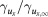, for the images of (a) Ketton, Mt Gambier, and ME2 and (b) Indiana, Estaillades, and ME1. The variograms are shown as a function of distance *x* normalized by the characteristic length *L*.

[24] The correlation structure is shown in Figure 5, where the variograms for porosity, 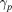, and velocity in the direction of flow, 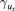, for the images of (a) Ketton, Mt Gambier, and ME2 and (b) Indiana, Estaillades, and ME1 are plotted. The functions are calculated as

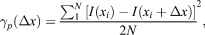
(4)

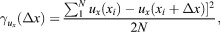
(5)
where 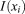 is the indicator function for porosity (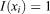 for pore voxels and 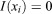 for grain voxels), 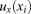 are velocities in the direction of flow across faces oriented normal to the *x* direction, and *N* is the number of voxels. Plotted are the 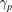 and 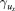 values normalized to the theoretical values at infinite range (uncorrelated limit) 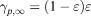 and 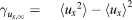. The *x* axis values are normalized to the characteristic length *L* estimated for each carbonate sample: the values for *L* are presented in Table1.

[25] The variograms for porosity indicate a correlation length (the distance when the variogram reaches its maximum-sill-value) that is approximately the characteristic length—a typical grain size, although it is larger than this in some cases, particularly for Estaillades. The correlation length for the velocity field is, in general, greater than for porosity, especially for the more heterogeneous samples. Mt Gambier also has a large apparent correlation, but its characteristic length (see Table1) is less than half of that for the other samples, while the approach to the sill is smooth, unlike the more structured correlation displayed by the heterogeneous samples in Figure 5b. The correlation length for Mt Gambier (measured in µm) is comparable with the other homogeneous samples.

**Figure 6:**
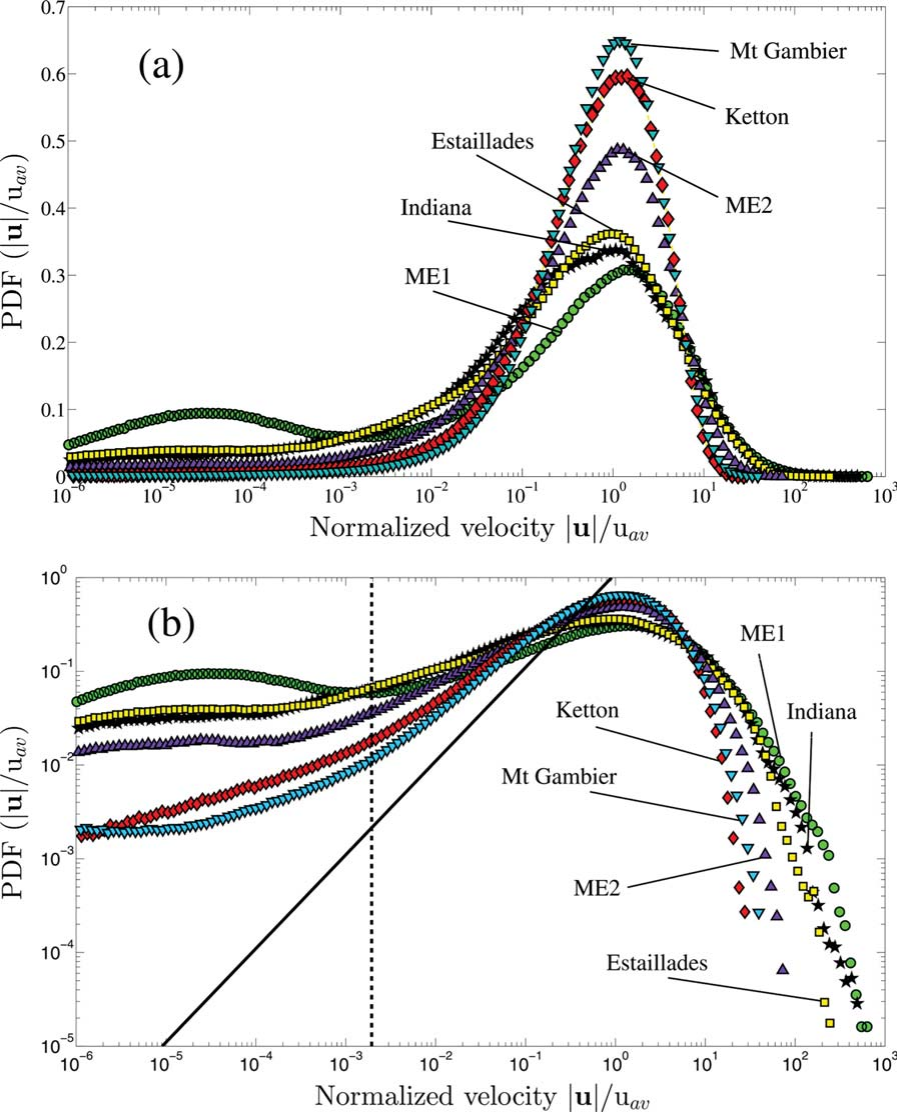
PDF of the velocity distributions for the carbonates studied presented as (a) semilog and in (b) log-log plot. The dashed line indicates a diffusive cutoff for which the time taken to traverse a voxel by advection is 100 times longer to that traversed by diffusion for a base-case Peclet number of 200. Velocities lower than this are practically not sampled since the solute diffuses out of these stagnant regions. The solid line shows the velocity distribution in a single circular tube, representing the homogeneous limit.

[26] In Figure 6, PDFs of the ratio of the magnitude of ***u*** (at the voxel centers) divided by the average flow speed *u*_av_ are presented as semilog and log-log plots. The PDFs are calculated as histograms of the velocity distributions sampled uniformly in 256 bins of log(|***u***|/*u*_av_). For reference on the same plots we present the homogeneous limit: the analytical PDF of |***u***|/*u*_av_ for a single circular cylindrical tube. The PDFs of |***u***|/*u*_av_ exhibit different characteristics in terms of the spread between low and high velocities, and the magnitude of the peak centered on |***u***|/*u*_av_ = 1. It is evident from Figure 6a that in all the carbonate samples many velocities are many orders of magnitude lower than the average flow speed, while the values for higher velocities show different spreads. We will use these characteristics to interpret the shapes of dispersion propagators in section 3 that explain the origin of early breakthrough and long tailing plume behavior.

[27] Indicated on Figure 6b by the vertical line is the velocity *u*_min_ at which the time taken to traverse a voxel of size Δ*x* by advection 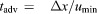 is 100 times longer to that traversed by diffusion 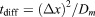 for a base-case Peclet number of 200. The base-case value for molecular diffusion coefficient *D_m_* is 1.5 × 10^−9^ m^2^/s which for Indiana limestone yields *Pe* = 200 (using the characteristic length of *L* = 0.2996 mm and *u*_av_ = 1 mm/s). Smaller velocities are unlikely to have much impact on transport in these regions of the pore space since diffusion will dominate. As we discuss later, for larger *Pe* this limit is shifted to smaller velocities. Note that there are always a significant number of very small speeds, indicating that some diffusion is necessary to allow solute to move throughout the pore space.

### 2.4. Transport Model

[28] We simulate transport by moving an ensemble of particles by advection along streamlines, using a semianalytic description of the velocity field within a grid block for all combinations of solid boundaries [*Mostaghimi et al*., 2012]. A random walk method is used to describe molecular diffusion: a particle instantaneously jumps over a mean free path 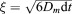 in a random direction. The time step d*t* for all simulations is 10^−4^ s, and it does not change with *Pe*; the average motion of particle at each time step is less than one voxel. We change *Pe* by varying *D_m_*. Particles are placed throughout the image volume in uniformly spaced voxels; within each voxel the particle is placed at random. The number of particles ranges from 1,000,000 to 2,000,000. We apply a reflection boundary condition for the particles that hit the surface of the solid voxels. If a particle exits the inlet or outlet face of the cubic image, it is randomly reassigned to the opposite face: flux-weighted during the advective step and area-weighted for the diffusive step [*Bijeljic et al*., 2004]. Reflecting boundary conditions are used for the other image faces.

[29] We track particles and plot concentration profiles as a function of particle displacements (propagators). Propagators are calculated such that 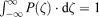, where *P*(*ζ*) is the probability of particle displacement *ζ*. The propagator is the probability that a particle has moved a distance *ζ* in the main flow direction and is equivalent to the concentration profile resulting from an initial delta-function pulse (mathematically, the Green function for the transport).

## 3. Transport Results

[30] First, we study the impact of structure on the nature of early breakthrough and long tailing plume behavior by analyzing displacement probabilities (propagators) on our suite of carbonate images for *Pe* = 200. Second, we extend our study to examine the impact of Peclet number. For both parts, we use the velocity distributions from Figure 6 to explain the behavior.

**Figure 7:**
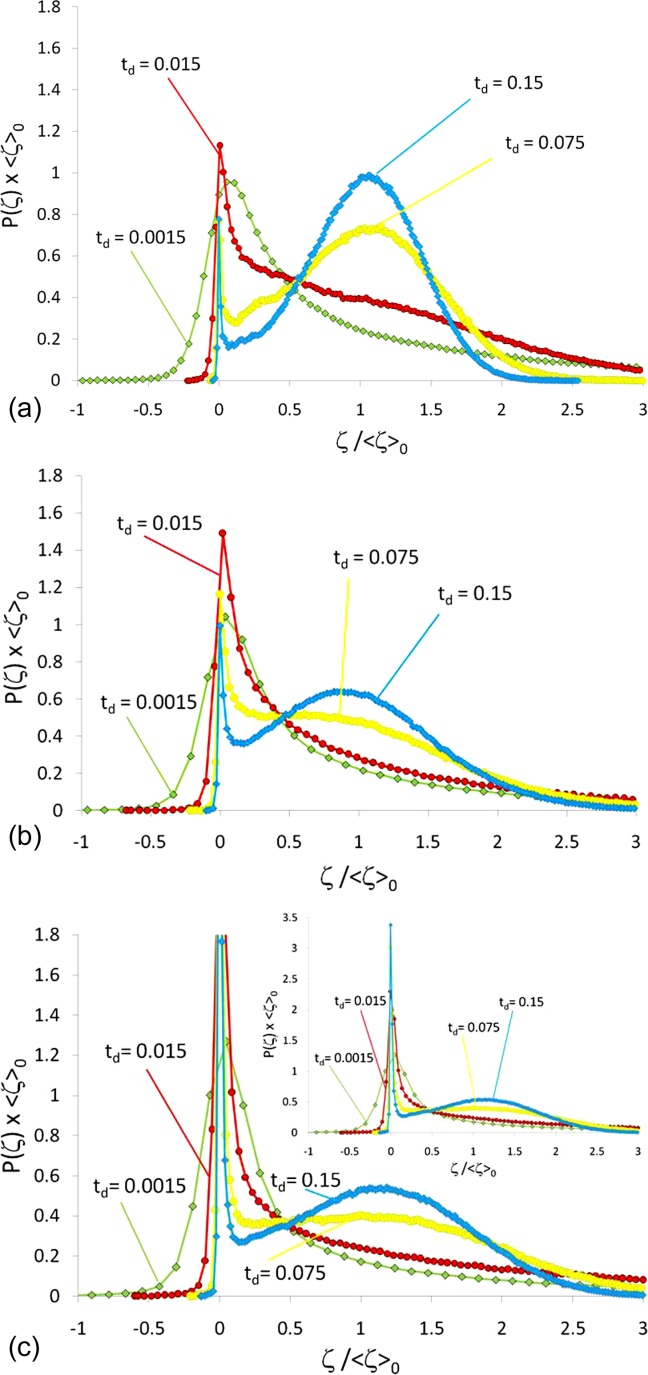
Probability of molecular displacement *P*(*ζ*) in the images of (a) Ketton limestone, (b) Mt Gambier limestone, and (c) ME2 as a function of displacement in the flow direction ζ for the set of times *t_d_* = 0.0015, 0.015, 0.075, and 0.15. The coordinates are rescaled by the expected nominal mean displacement 〈*ζ*〉_0_ = *u*_av_*t* in the direction of flow. *Pe* = 200. The insert in (c) shows larger scale for *P*(*ζ*)×*〈ζ*〉_0_.

### 3.1. Non-Fickian Propagators in Carbonates: Impact of Pore Structure

[31] We first present the evolution of the propagators relative to the expected mean displacement in the main flow direction in carbonates with a relatively narrow spread of velocities (as shown in Figure 6): Ketton (Figure 7a), Mt Gambier (Figure 7b), and ME2 (Figure 7c). These will be later compared to the propagators in carbonates with a wider spread of velocities.

[32] We define a dimensionless time 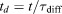, where 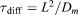. This is the ratio of the time to the time to traverse a characteristic length by diffusion. In this paper we study preasymptotic, non-Fickian transport where *t_d_* < 1; only for *t_d_* >> 1 does Fickian behavior emerge, once diffusion has allowed the solute to sample to entire flow field [*Salles et al*., 1993]. Dimensionless time *t_d_* multiplied by the Peclet number represents the number of characteristic lengths the solute has traveled on average. Our focus is on *t_d_* < 1 but where *t_d_Pe* > 1.

**Figure 8:**
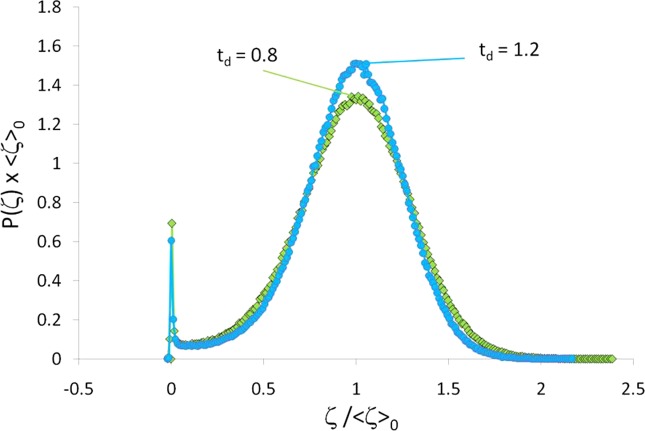
Probability of molecular displacement *P*(*ζ*) in the Mt Gambier image as a function of displacement *ζ* for times *t_d_* = 0.8 and 1.2. The coordinates are rescaled by the expected nominal mean displacement 〈*ζ*〉_0_ = *u*_av_*t* in the direction of flow. *Pe* = 200.

[33] Probabilities of displacement are plotted in Figure 7 for dimensionless times *t_d_* = 0.0015, 0.015, 0.075, and 0.15 for *Pe* = 200. At early times (*t_d_* = 0.0015), due to a significant portion of fluid residing in stagnant zones for which diffusion is the main mechanism of transport, the solute can move against the main flow direction (a negative displacement). There is a concentration peak of stagnant solute centered around zero, while the flowing solute has an elongated moving tail with no pronounced mobile peaks. As time progresses, more and more particles diffuse out of the stagnant regions, which results in the stagnant peak becoming narrower with time on rescaled distance axes, while there is a formation of a secondary mobile solute peak in concentration that becomes prominent around *t_d_* = 0.075 and dominates at later times (*t_d_* = 0.15). This reflects the particles that eventually diffuse out from the slow-moving regions and then move rapidly through the better connected, wider regions. The diffusion time for the particles to diffuse a characteristic length (say, the distance between pores) is *t_d_* = 1. This is 14.4 s for Mt Gambier limestone, 32.3 s for ME2, and 64.1 s for Ketton limestone. The emergence of Gaussian behavior can be seen in Figure 8, where propagators for Mt Gambier are plotted for *t_d_* = 0.8 and *t_d_* = 1.2. The mobile peak in solute concentration increases with time and almost entirely dominates the slow-moving region that is gradually disappearing at longer times.

**Figure 9:**
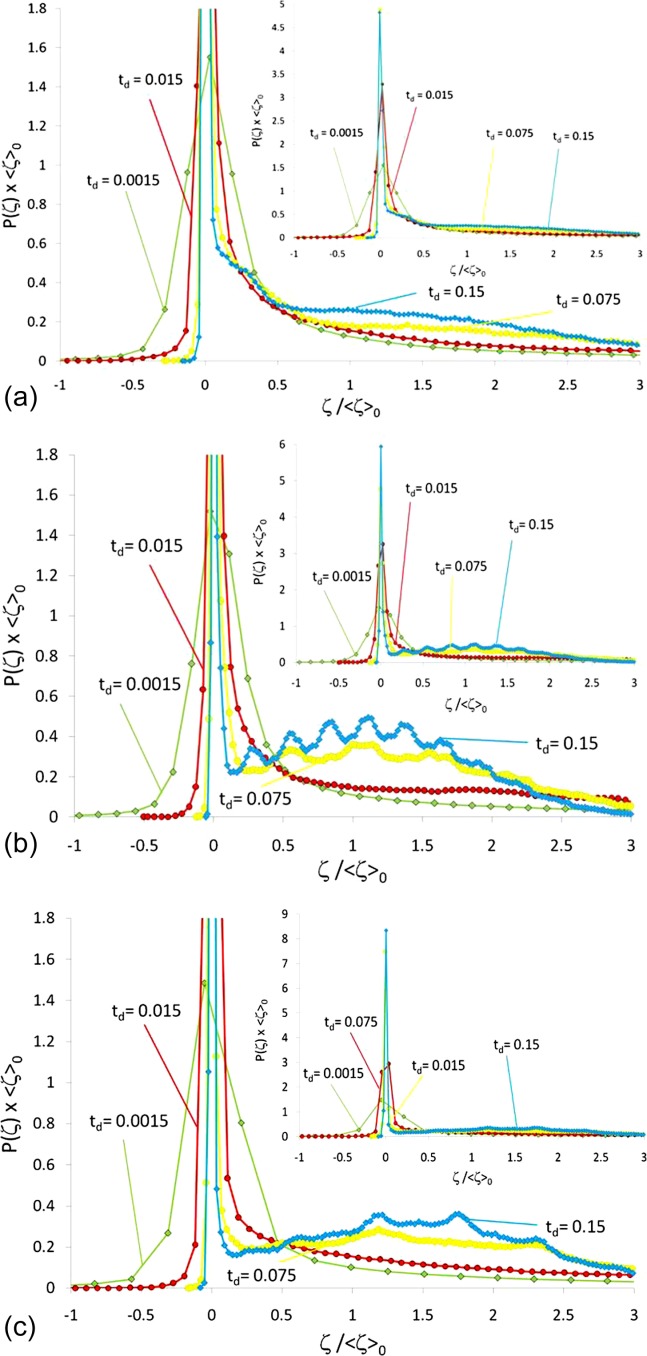
Probability of molecular displacement *P*(*ζ*) in images of (a) Estaillades limestone, (b) Indiana limestone, and (c) ME1 as a function of displacement in the flow direction *ζ* for the set of times *t_d_* = 0.0015, 0.015, 0.075, and 0.15. The coordinates are rescaled by the expected nominal mean displacement 〈*ζ*〉_0_ = *u*_av_*t* in the direction of flow. *Pe* = 200. The inserts show larger scale for *P*(*ζ*)*×〈ζ*〉_0._

[34] More persistent non-Fickian behavior is observed for the carbonates with a wide spread of velocities. Figure 9 show the propagators for Estaillades limestone, Indiana limestone, and ME1, where large, long-lasting concentration peaks of stagnant fluid are seen. This indicates that more solute is retarded in diffusion-dominated zones, while less is free to flow through connected channels, resulting in an elongated plume tail at early times and a smaller mobile peak at later times (*t_d_* = 0.15).

[35] The characteristic time to diffuse out of a single stagnant pore is similar in these cases, and yet the approach to Gaussian-like behavior is slower than in the less heterogeneous samples. This indicates that there is correlated heterogeneity in the flow field, as indicated in Figure 5, meaning that to reach a fast-flowing domain, particles have to diffuse through several stagnant pores, giving a much larger timescale to see the emergence of approximately Gaussian behavior.

**Figure 10:**
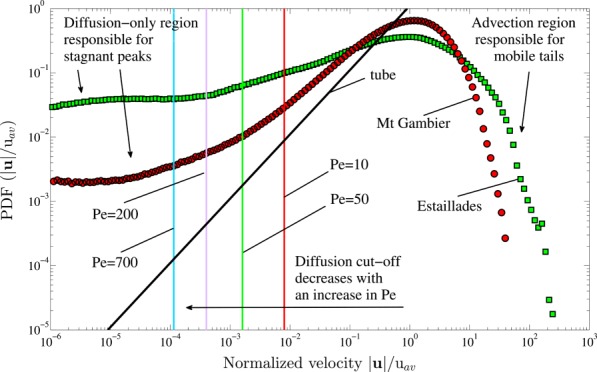
Voxel velocity distributions for Mt Gambier limestone and Estaillades limestone. Mt Gambier limestone has a narrow spread of velocities, while Estaillades limestone has a wide spread of velocities. This results in a different shape of propagators (stagnant concentration regions, elongated mobile tails) and hence the different nature of transport. The lines indicate diffusive cutoffs at *Pe* = 10, 50, 200, and 700 for which the time taken to traverse a voxel by advection is 100 times longer to that traversed by diffusion for Mt Gambier limestone. The arrow shows that diffusive cutoff decreases with an increase in *Pe*. The solid line shows the velocity distribution in a single circular tube, representing the homogeneous limit.

[36] We can explain the complex non-Fickian transport behavior of propagators described in Figures 9 by looking at the velocity distribution curves in Figure 10. The exemplars taken are Mt Gambier limestone for a narrow spread of velocities and Estaillades limestone for a wide spread. Note that they are different in both low-velocity regions where diffusion is the only mechanism of transport leading to largely immobile solute, and the high-velocity region that produces the elongated tail of fast-moving solute. These characteristics define the nature of transport revealed by the different shapes of the propagators.

[37] In the carbonates with a narrow spread of velocities (Ketton and Mt Gambier and ME2 in Figure 7), transport is characterized by a smaller immobile concentration and a significantly larger secondary peak in mobile tracer concentration. On the other hand, in samples with a wider spread of velocity (Estaillades, Indiana, and ME1 in Figure 9), transport is characterized by a significant immobile concentration and an elongated tail of fast-moving solute.

[38] The generic transport behavior can be predicted from the velocity distribution (Figures 6 and 10), pore size distribution (Figure 2), and the connectivity combined with the velocity field (Figures 3 and 4). While a lower porosity and connectivity with a wide spread of velocities result in most anomalous transport (Estaillades, Indiana, and ME1), a higher porosity and connectivity and a narrow spread of velocities result in less anomalous transport behavior (Ketton, Mt Gambier, and ME2).

**Figure 11:**
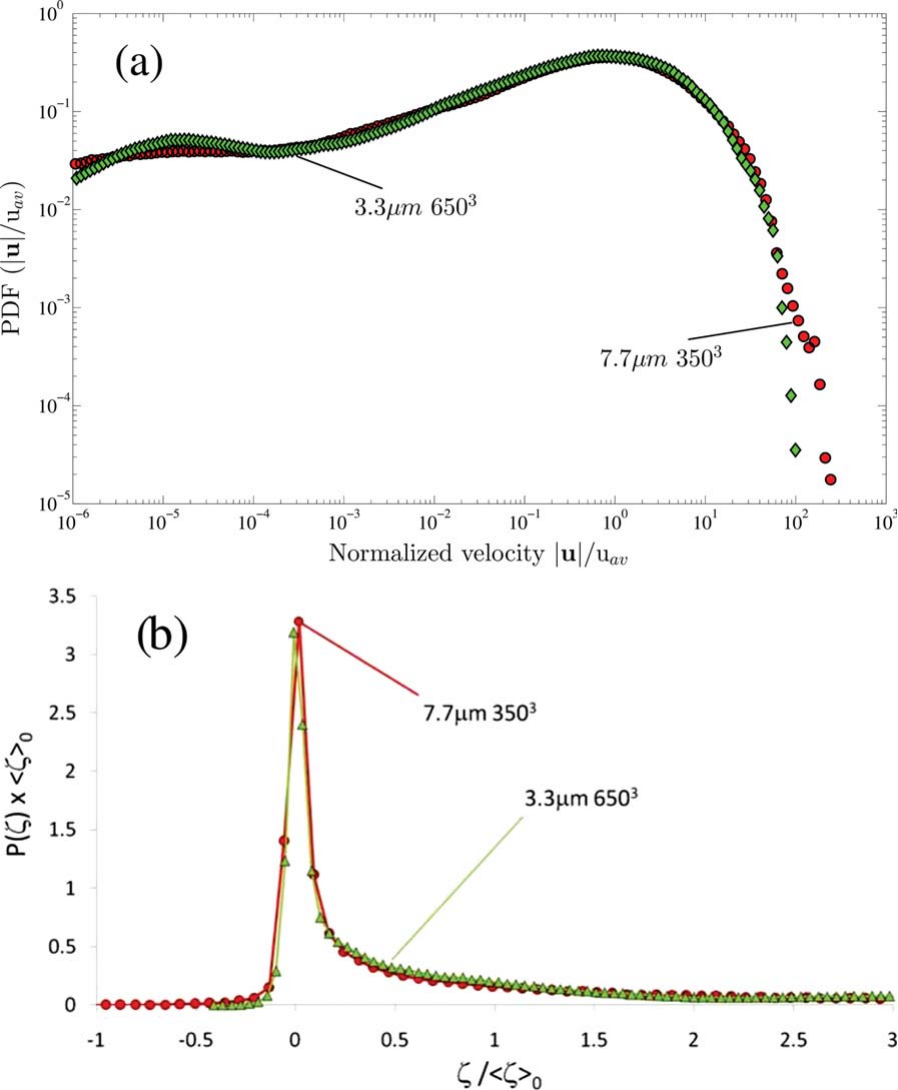
(a) Voxel velocity distributions and (b) probability of molecular displacements *P*(*ζ*) for time *t_d_* = 0.015 for two images of Estaillades limestone. Computations on a 350^3^ image with a voxel size of 7.7 µm (labeled Estaillades in Table1) are compared to the results from a 650^3^ image with a voxel size of 3.3 µm (Estaillades high resolution in Table1).

[39] We explore the effect of image resolution in Figures 11a and 11b where we compare the velocity fields and propagators for Estaillades for the 350^3^ image with a voxel size of 7.7 µm and the 650^3^ Estaillades higher-resolution image with a voxel size of 3.3 µm. The velocity fields are virtually identical with, perhaps, more slow-flowing regions identified in the higher-resolution image. There is very little difference in the predicted propagators. Improving the image resolution allows more of the pore space to be captured, although there is still unresolved microporosity. However, there is, with finite computational resources, a trade-off between resolution and total system size. One cannot both resolve microporosity and run simulations on an image that spans several characteristic lengths, and which is therefore representative of core-scale transport.

[40] The complex non-Fickian transport behavior of propagators described has significant implications for mixing and large-scale transport. In order to describe long tailing plume behavior from the core scale, the plume retardation arising from stagnant flow regions needs to be incorporated, while the early breakthrough behavior needs to account for secondary mobile peaks. This requires, as discussed in section 1, a more sophisticated large-scale transport model, such as those based on CTRW or multirate transfer models [*Haggerty and Gorelick*, 1995; *Haggerty et al*., 2000; *Berkowitz et al*., 2006].

### 3.2. Dependence on Peclet Number

[41] We study the effect of *Pe* on transport by taking exemplars representing the two generic types of behavior mentioned previously: Mt Gambier and Estaillades limestone.

**Figure 12:**
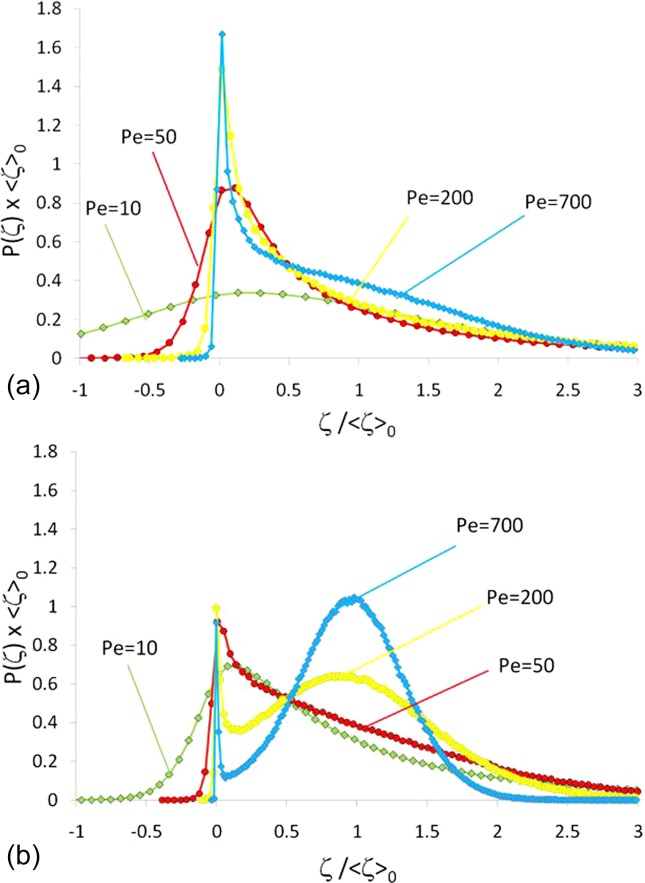
Probability of molecular displacement *P*(*ζ*) in images of Mt Gambier limestone as a function of displacement in the flow direction *ζ* for different *Pe* = 10, 50, 200, and 700 at times (a) *t_d_* = 0.015 and (b) *t_d_* = 0.15. The coordinates are rescaled by the nominal mean displacement 〈*ζ*〉_0_ = *u*_av_*t*.

[42] Figures 12a and 12b compare the propagators for Mt Gambier at dimensionless times *t_d_* = 0.015 and *t_d_* = 0.15, for *Pe* =10, 50, 200, and 700. At early times (*t_d_* = 0.015) lower *Pe* leads to a more diffusive transport with displacement centered on zero. On the other hand, the fast flow in mobile zones is more pronounced at higher *Pe*, where advection is more important, leading to the faster formation of the secondary mobile peaks (as seen for *t_d_* = 0.15).

**Figure 13:**
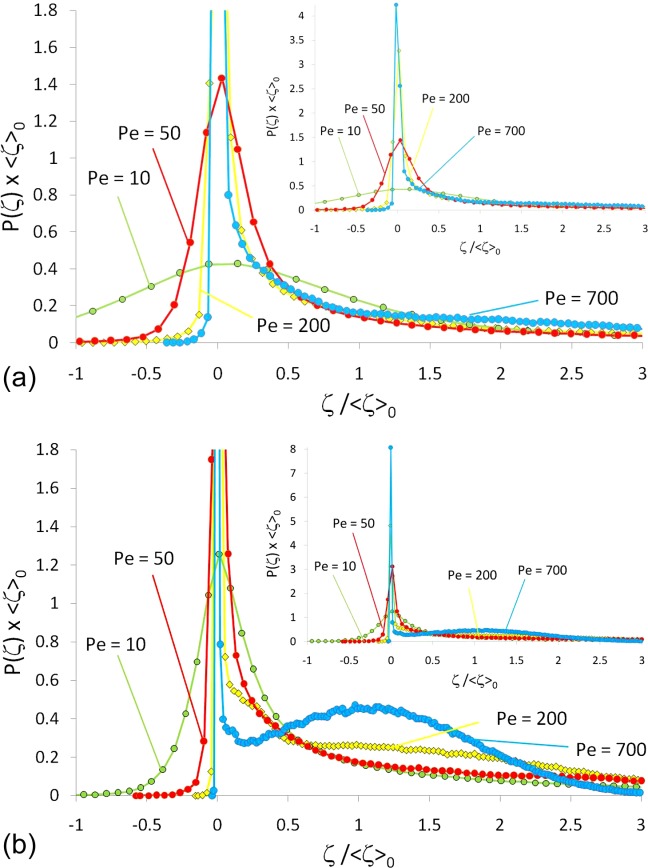
Probability of molecular displacement *P*(*ζ*) in images of Estaillades limestone as a function of displacement in the flow direction *ζ* for different *Pe* = 10, 50, 200, and 700 at times (a) *t_d_* = 0.015 and (b) *t_d_* = 0.15 (b). The coordinates are rescaled by the nominal mean displacement 〈*ζ*〉_0_ = *u*_av_*t*.

[43] The impact of *Pe* on the propagators for Estaillades is presented in Figures 13a and 13b for the same set of dimensionless times and *Pe* as in the case of Mt Gambier. Immobile fluid regions are seen for both times and all *Pe*. For late times (*t_d_* = 0.15) the formation of mobile peak is seen at highest *Pe* =700, although even in this case the persistent stagnant region is still present.

[44] The impact of *Pe* on the shape of the propagators can be analyzed by looking at the velocity distributions. In Figure 10 we plotted diffusive cutoffs at *Pe* = 10, 50, 200, and 700 for which the time taken to traverse a voxel by advection is 100 times longer to that traversed by diffusion for Mt Gambier limestone. This means that essentially the only transport mechanism for these voxels is diffusion. With an increase in *Pe*, the diffusive cutoff moves to a lower value resulting in fewer voxels for which diffusion is the dominant mechanism of transport. Hence, as we vary *Pe* (either the overall flow rate or diffusion coefficient), the sampling of the velocity distribution changes. Thus, the diffusion-controlled stagnant regions of concentration are less pronounced at higher *Pe*, as shown in Figures 12 and 13.

## 4. Discussion and Conclusions

[45] The transport behavior in carbonates is characterized by a stagnant peak concentration and a long fast-moving tail, controlled by the relative impact of diffusion and advection coupled to a wide range of flow velocities in a heterogeneous pore space.

[46] In carbonates with a wide pore size distribution coupled with a low connectivity that consequently exhibit a wide distribution of velocities, the peak plume position is retarded relative to the mean flow field with a very wide spread. There is an effectively immobile peak concentration and an elongated tail of fast-moving solute, characterized by secondary peaks in the mobile plume concentration. This new explanation is consistent with other studies of transport from the pore to the field scales in heterogeneous media [see, for instance, *Berkowitz et al*., 2006; *Gouze et al*., 2008b]. For the carbonates where the impact of structure (i.e., a narrow pore size distribution and/or a highly connected pore space) results in a narrow distribution of velocities, quantitatively different non-Fickian behavior is observed, as the concentration peak of stagnant fluid is much smaller. There is an elongated plume at early times and a single mobile peak moving at the average flow speed at later times. This behavior of propagators has significant implications for mixing, physical and chemical reaction, and large-scale transport: in order to describe long tailing plume behavior, the plume retardation arising from stagnant flow regions needs to be incorporated, while accounting for the early breakthrough with secondary single or multiple mobile peaks. This implies that simple average values for transport and reaction parameters, based on a Fickian formulation at the core scale, cannot be used for accurate upscaling in geological media with multiple heterogeneity scales. Appropriate approaches to deal with multiple-scale heterogeneity, from the pore scale upward, have been discussed by *Berkowitz et al*. [2008] and *Bijeljic et al*. [2011b].

[47] We have provided the evolution of propagators for different carbonates as a function of Peclet number and quantified the impact of flow rate and diffusion on the nature of non-Fickian transport. These can, in principle, be used in a larger-scale simulation of transport without the need to presume a governing transport equation. The propagator *P*(***x***,*t*;*Pe*) is simply the Green function for displacement. As a consequence, the concentration profile can formally be computed from

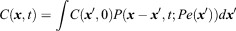
(6)
for an arbitrary initial concentration *C*(***x***,0). In a numerical simulation, permeability heterogeneity at, say, the core (cm) scale could be computed to find a flow field. This then defines a locally varying Peclet number *Pe*(***x***). If we have characterized the propagators as a function of *Pe*, then equation (6) allows the time evolution of an arbitrary initial plume to be computed in a domain that is heterogeneous at the core-to-field scale. Further details of a possible approach to this problem using a generalized network analysis and a CTRW approach can be found in *Rhodes et al*. [2008]. The development of this methodology is a topic for future work.
